# JAK inhibition during the early phase of SARS-CoV-2 infection worsens kidney injury by suppressing endogenous antiviral activity in mice

**DOI:** 10.1152/ajprenal.00011.2024

**Published:** 2024-04-18

**Authors:** Hibiki Sakai, Hiroyasu Kamuro, Nagisa Tokunoh, Takeshi Izawa, Shigeyuki Tamiya, Ayaha Yamamoto, Shota Tanaka, Daisuke Okuzaki, Chikako Ono, Yoshiharu Matsuura, Yoshiaki Okada, Yasuo Yoshioka, Yasushi Fujio, Masanori Obana

**Affiliations:** ^1^Laboratory of Clinical Science and Biomedicine, Graduate School of Pharmaceutical Sciences, Osaka University, Osaka, Japan; ^2^Vaccine Creation Group, BIKEN Innovative Vaccine Research Alliance Laboratories, Research Institute for Microbial Diseases, Osaka University, Osaka, Japan; ^3^Research Foundation for Microbial Diseases, Osaka University, Osaka, Japan; ^4^Laboratory of Veterinary Pathology, Osaka Metropolitan University Graduate School of Veterinary Science, Osaka, Japan; ^5^Department of Microbiology and Immunology, School of Pharmaceutical Sciences, Wakayama Medical University, Wakayama, Japan; ^6^Genome Information Research Center, Research Institute for Microbial Diseases, Osaka University, Osaka, Japan; ^7^Laboratory of Virus Control, Research Institute for Microbial Diseases, Osaka University, Osaka, Japan; ^8^Center for Infectious Disease Education and Research, Osaka University, Osaka, Japan; ^9^Center for Advanced Modalities and DDS, Osaka University, Osaka, Japan; ^10^Institute for Open and Transdisciplinary Research Initiatives, Osaka University, Osaka, Japan; ^11^Global Center for Medical Engineering and Informatics, Osaka University, Osaka, Japan; ^12^Laboratory of Nano-Design for Innovative Drug Development, Graduate School of Pharmaceutical Sciences, Osaka University, Osaka, Japan; ^13^Radioisotope Research Center, Institute for Radiation Sciences, Osaka University, Osaka, Japan

**Keywords:** antiviral activity, baricitinib, JAK, kidney injury, SARS-CoV-2

## Abstract

Coronavirus disease 2019 (COVID-19) induces respiratory dysfunction as well as kidney injury. Although the kidney is considered a target organ of severe acute respiratory syndrome coronavirus 2 (SARS-CoV-2) and affected by the COVID-19-induced cytokine storm, the mechanisms of renal reaction in SARS-CoV-2 infection are unknown. In this study, a murine COVID-19 model was induced by nasal infection with mouse-adapted SARS-CoV-2 (MA10). MA10 infection induced body weight loss along with lung inflammation in mice 4 days after infection. Serum creatinine levels and the urinary albumin/creatinine ratio increased on *day 4* after MA10 infection. Measurement of the urinary neutrophil gelatinase-associated lipocalin/creatinine ratio and hematoxylin and eosin staining revealed tubular damage in MA10-infected murine kidneys, indicating kidney injury in the murine COVID-19 model. Interferon (IFN)-γ and interleukin-6 upregulation in the sera of MA10-infected mice, along with the absence of MA10 in the kidneys, implied that the kidneys were affected by the MA10 infection-induced cytokine storm rather than by direct MA10 infection of the kidneys. RNA-sequencing analysis revealed that antiviral genes, such as the IFN/Janus kinase (JAK) pathway, were upregulated in MA10-infected kidneys. Upon administration of the JAK inhibitor baricitinib on *days 1*–*3* after MA10 infection, an antiviral pathway was suppressed, and MA10 was detected more frequently in the kidneys. Notably, JAK inhibition upregulated the hypoxia response and exaggerated kidney injury. These results suggest that endogenous antiviral activity protects against SARS-CoV-2-induced kidney injury in the early phase of infection, providing valuable insights into the pathogenesis of COVID-19-associated nephropathy.

**NEW & NOTEWORTHY** Patients frequently present with acute kidney injury or abnormal urinary findings after severe acute respiratory syndrome coronavirus 2 (SARS-CoV-2) infection. Here, we investigated how the kidneys respond during SARS-CoV-2 infection using a murine coronavirus disease 2019 (COVID-19) model and showed that Janus kinase-mediated endogenous antiviral activity protects against kidney injury in the early phase of SARS-CoV-2 infection. These findings provide valuable insights into the renal pathophysiology of COVID-19.

## Graphical abstract

**Figure:**
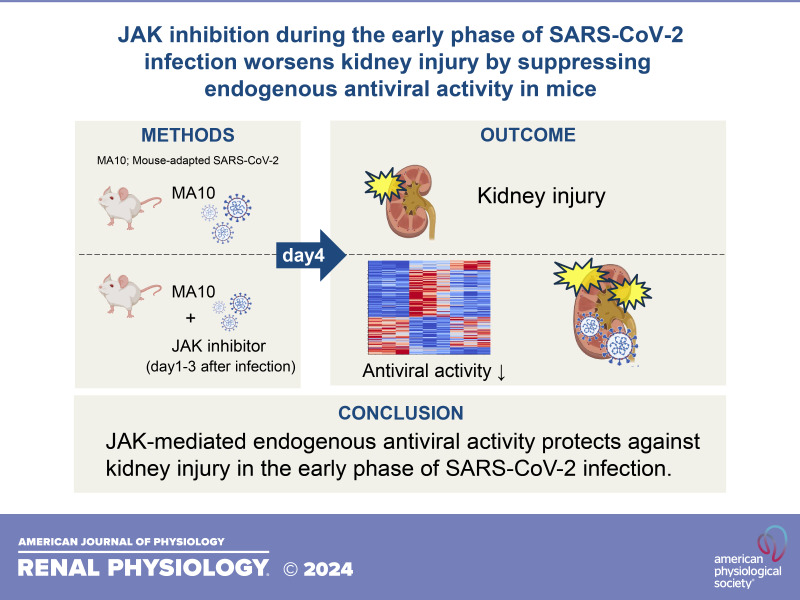

## INTRODUCTION

Although the main symptom of coronavirus disease 2019 (COVID-19) is respiratory dysfunction, kidney injury is recognized as a consequential complication of severe acute respiratory syndrome coronavirus 2 (SARS-CoV-2) infection (1). Patients often present with acute kidney injury (AKI) or abnormal urinary findings, including glomerular diseases, after SARS-CoV-2 infection (2, 3). SARS-CoV-2 enters target cells through angiotensin-converting enzyme (ACE) 2, which is modulated by disintegrin and metalloprotease domain-containing proteins 10 and 17, and transmembrane protease serine 2 (4, 5). Beyond the lung, ACE2 is expressed in the kidney, particularly in tubular epithelial cells and podocytes (6, 7). Moreover, as SARS-CoV-2 is detected in the urine of patients with COVID-19 (8, 9), the kidney is considered a target organ of SARS-CoV-2; however, SARS-CoV-2 is not observed in kidney biopsies from patients with COVID-19 (10–12). Thus, the mechanisms behind the SARS-CoV-2 infection-induced disruption of renal homeostasis, whether through direct viral invasion or the indirect effects of a cytokine storm and cardiocirculatory failure, remain elusive.

Pharmacotherapy against COVID-19 has seen numerous advances. Currently, patients with mild and moderate COVID-19 symptoms receive antiviral therapies and neutralizing monoclonal antibodies (13). Immunosuppression therapies, such as steroids, anti-interleukin (IL)-6 receptor antibodies, and Janus kinase (JAK) inhibitors, are used for the treatment of patients with severe COVID-19 symptoms in the late stage of SARS-CoV-2 infection. Interferon (IFN)-α, IFN-β, and IFN-γ, which activate JAK1/2, are upregulated in patients with COVID-19 (14, 15). A recent study revealed that JAK inhibitors block the COVID-19 cytokine-induced injury in human organoid-derived podocytes (16), suggesting a therapeutic avenue against kidney injury in COVID-19. Furthermore, IFNs exhibit antiviral activities against RNA viruses, including SARS-CoV-2. Considering the timing of administration of JAK inhibitors, that JAK inhibitors are used in combination with an antiviral drug remdesivir, and that SARS-CoV-2 infection is less likely to occur in the kidneys, we hypothesize that the kidneys are resistant to injury because of antiviral activity during the early stage of COVID-19.

In this study, we focus on kidney injury because renal dysfunction is associated with disease severity and poor short-term prognosis in patients with COVID-19 (17). The aim of this study is to examine how renal homeostatic mechanisms function in SARS-CoV-2 infection by analyzing the effects of a JAK inhibitor, baricitinib, on the kidneys of an in vivo murine COVID-19 model. Specifically, we induce a murine COVID-19 model by nasal infection with mouse-adapted SARS-CoV-2 (MA10), which then induces lung and kidney injury. According to our results, MA10 is not preferentially detected in the kidneys of MA10-infected mice, and cytokines, including IFN-γ and IL-6, are upregulated in the sera of MA10-infected mice, suggesting that cytokine signaling may affect renal homeostasis in COVID-19. Intriguingly, baricitinib treatment on *days 1*–*3* after MA10 infection exaggerates kidney injury by suppressing intrinsic antiviral activity and increasing the hypoxia response. These findings provide a valuable perspective on the pathogenesis of COVID-19-associated nephropathy.

## MATERIALS AND METHODS

### Infection With Mouse-Adapted SARS-CoV-2

MA10 containing seven mutations in the SARS-CoV-2 NIID strain obtained from the Research Institute for Microbial Diseases (nsp4: T295I, nsp7: K2R, nsp8: E23G, S: Q493K/Q498Y, P499T, and orf6: F7S) was induced using the circular polymerase extension reaction method, as previously described (18, 19). Male BALB/c mice (6–10 wk old) were intranasally administered 2 × 10^5^ plaque-forming units (PFU)/mouse MA10. Spot urine samples were collected from the mice. At the end of all experiments, the mice were deeply anesthetized with isoflurane (Fuji Film Wako, Tokyo, Japan) or anesthetic mixed with medetomidine (ZENOAQ, Koriyama, Japan, 0.3 mg/kg), midazolam (Maruishi Pharmaceutical, Osaka, Japan, 4.0 mg/kg), and butorphanol (Meiji Seika Pharma, Tokyo, Japan, 0.3 mg/kg) to minimize suffering, and their blood, lungs, and kidneys were collected.

### Treatment of Mice

The JAK inhibitor baricitinib was purchased from Selleck Chemicals (Houston, TX). Mice were orally administered 2 mg/kg (low dose) or 10 mg/kg (high dose) baricitinib or 0.5% carboxymethyl cellulose (CMC) as a vehicle on *days 1*–*3* after the MA10 infection.

### Hematoxylin and Eosin Staining

The lungs and kidneys were collected from MA10-infected mice and fixed with 10% neutral buffered formalin for over 3 days. Paraffin sections (3 µm) were stained with hematoxylin and eosin. Images were captured using the BZ-X700 system (Keyence, Osaka, Japan) or the BX53 microscope (Evident, Tokyo, Japan) with a DS-Fi1 camera system (Nikon, Tokyo, Japan). Histopathological examination of the lungs and kidneys was performed by a veterinary pathologist (T.I., diplomate of the Japanese Society of Toxicologic Pathology, and diplomate of the Japanese College of Veterinary Pathologists). Briefly, the severity of inflammation in the alveolus/alveolar wall, regeneration of the bronchiolar epithelium, hemorrhage within the alveolar spaces, and fibrin exudation or accumulation of proteinaceous eosinophilic materials within the alveoli were scored from 0 to 3 (0: absent; 1: mild; 2: moderate; and 3: severe). The lung severity score was calculated as the average of all four evaluation items. Morphological abnormalities were not observed in the glomeruli of most mice; therefore, the degeneration of tubular cells (swelling of tubular cells, vacuolation, swelling of nuclei, and decreased chromatin density) was evaluated using scores ranging from 0 to 3 (0: absent; 1: mild; 2: moderate; and 3: severe).

### Immunohistochemistry

Paraffin sections (3 µm) of kidneys were stained with antiangiopoietin-like (ANGPTL) 4 (Invitrogen/Thermo Fisher Scientific, No. 40-9800, Waltham, MA) antibody using VECTASTAIN ABC kit (Vector Laboratories, Burlingame, CA). The nuclei were stained with hematoxylin. Images were captured using the BZ-X700 system (Keyence) and quantification was performed by a researcher who was blinded to the experimental group.

### PCR/Quantitative PCR Analysis

Total RNA was prepared from murine lungs or kidneys using QIAzol Lysis Reagent (Qiagen, Hilden, Germany). Because preliminary studies showed that tubular cells not only at the cortex but also at the outer medulla were affected by MA10 infection, total RNA was extracted from the entire kidney. Complementary DNA was synthesized using random primers (Invitrogen/Thermo Fisher Scientific) and ReverTra Ace (Toyobo, Osaka, Japan). PCR was performed using the AmpliTaq Gold 360 Master Mix (Applied Biosystems/Thermo Fisher Scientific) with specific primers for the detection of MA10. The PCR products were analyzed using agarose gel electrophoresis. Gene expression was quantified using a Fast SYBR Green Kit (Applied Biosystems/Thermo Fisher Scientific). The primers used in this study are shown in Supplemental Table S1.

### Measurement of Biochemical Parameters

The creatinine levels in sera and urine were measured using the LabAssay Creatinine Kit (FUJIFILM Wako Pure Chemical) according to the manufacturer’s protocol.

Urinary albumin and neutrophil gelatinase‐associated lipocalin levels (NGAL) were measured using the LBIS Mouse albumin ELISA Kit (FUJIFILM Wako Shibayagi, Gunma, Japan) and the Mouse Lipocalin-2/NGAL Quantikine ELISA Kit (R&D Systems, Minneapolis, MN), respectively. The urinary albumin-to-creatinine ratio (ACR) and NGAL/Cr ratio were also calculated.

IFN-γ, IL-6, and tumor necrosis factor (TNF)-α protein levels in mouse sera were measured using a Mouse IFN-γ Quantikine ELISA Kit (R&D Systems), a Mouse IL-6 Quantikine ELISA Kit (R&D Systems), and a Mouse TNF-α Quantikine ELISA Kit (R&D Systems), respectively. Aspartate aminotransferase levels in sera were measured using a Mouse AST ELISA Kit (Abcam, Cambridge, UK).

### RNA-Sequencing and Data Processing

Total RNA was extracted from the kidneys of noninfected and MA10-infected mice treated with vehicle (0.5% CMC) or high-dose baricitinib. Library preparation was conducted using TruSeq Stranded mRNA Library Prep (Illumina), with sequencing performed on an Illumina NovaSeq 6000 platform in a 101 base single read mode. The sequenced reads were aligned to mouse reference genome sequences (rn6) using TopHat version 2.1.1. Fragments per kilobase of exon per million mapped fragments were calculated using Cuffdiff version 2.2.1.

Differentially expressed genes (DEGs) between the noninfection and MA10 + vehicle groups were screened according to the following criteria: log_2_ fold change > 0.58 and *P* < 0.05. Regarding the genes whose expression varied with MA10 infection, DEGs between the MA10 + vehicle and MA10 + JAKi groups were screened using the same criteria. Significant DEGs were visualized using heat maps and volcano plots. Heatmaps and volcano plots were produced using the R packages heatmap and ggplot (v.4.3.1). Gene Ontology (GO) annotation of DEGs was performed using the Enrichr website database (https://maayanlab.cloud/Enrichr/). Gene names of the DEGs were imported into the website, and the top 10 functions or pathways were selected with scores based on a combination of *P* values and odds ratios.

### Statistical Analysis

Data are shown as the means ± SD. Student’s *t* test, Welch’s *t* test, or the Mann–Whitney *U* test were used for comparisons between two groups. One-way analysis of variance, followed by Dunnett’s test, was used for multiple comparisons. *P* < 0.05 was considered statistically significant.

### Study Approval

Animal care was performed in accordance with the Osaka University Animal Care Guidelines. All experimental procedures conformed to the National Institutes of Health *Guide for the Care and Use of Laboratory Animals* (8th ed.), which was updated by the United States National Research Council in 2011. All experimental protocols were reviewed and approved by the Animal Care and Use Committee of the Graduate School of Pharmaceutical Sciences, Osaka University (Approval No. Douyaku R01-1-6) and by the Research Institute for Microbial Diseases, Osaka University (Protocol Nos. BIKEN-AP-R01-15-2 and BIKEN-AP-R02-09-0). The mice were maintained in the Animal Care Facility of the Graduate School of Pharmaceutical Sciences or Research Institute for Microbial Diseases, Osaka University, under a 12:12-h light-dark cycle with ad libitum access to food and water. Experiments on mice infected with SARS-CoV-2 were performed in a biosafety level 3 facility at the Research Institute for Microbial Diseases, Osaka University. All experiments involving viruses were approved by the Institutional Review Board of the Research Institute for Microbial Diseases, Osaka University (Protocol Nos. BIKEN-04578, BIKEN-04624, and BIKEN-00137-047).

## RESULTS

### Kidney Injury Was Induced in MA10-Infected Mice

**Figure 1.**
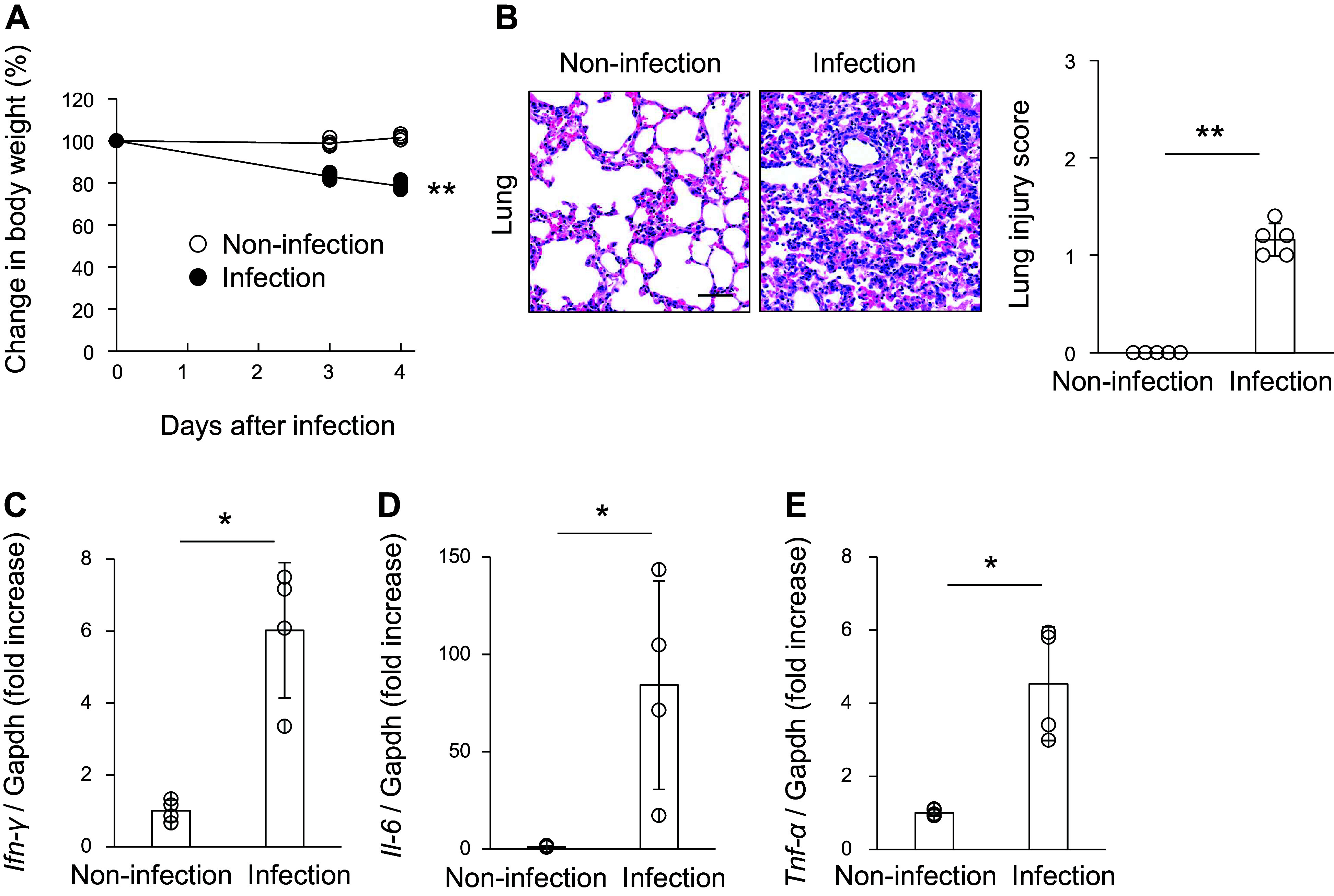
Intranasal injection of mouse-adapted severe acute respiratory syndrome coronavirus 2 (SARS-CoV-2) (MA10) resulted in lung injury. BALB/c mice were nasally infected with MA10. *A*: body weight changes measured after MA10 infection. Data are shown as means ± SD (*n* = 5); **P < 0.01 by Student’s *t* test. *B*: hematoxylin and eosin (H&E) staining of murine lungs after MA10 infection. Scale bar: 50 μm. The severity of lung injury scores is shown as means ± SD (*n* = 5); *P < 0.05 by Welch’s *t* test. *C*–*E*: expression of *Ifn-γ*, *Il-6*, and *Tnf-α* was examined on *day 4* after MA10 infection using quantitative PCR. Transcript expression was normalized to that of *Gapdh*. Data are shown as means ± SD (*n* = 4–5); *P < 0.05 and **P < 0.01 by Welch’s *t* test or Mann–Whitney *U* test.

**Figure 2.**
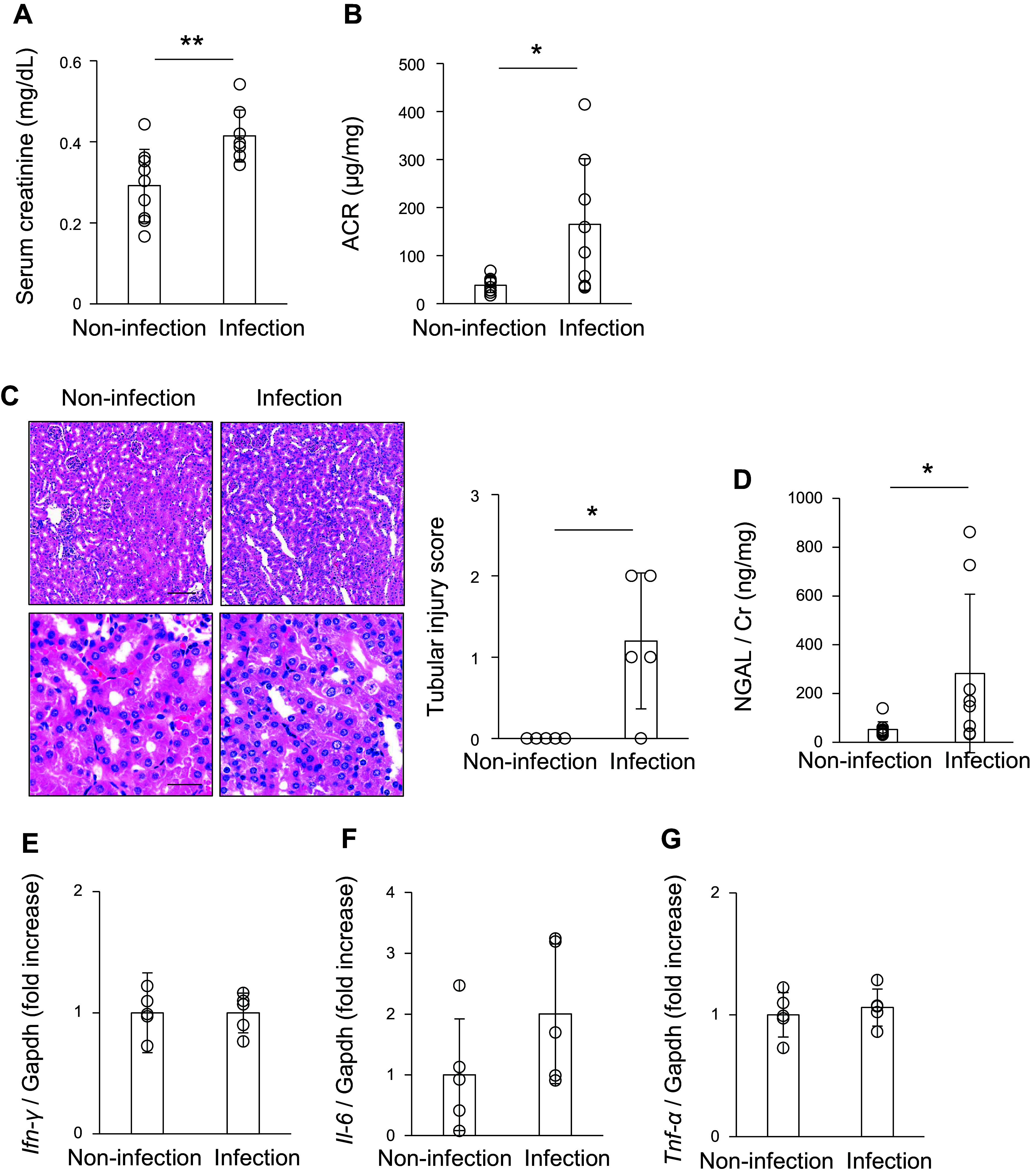
Intranasal injection of mouse-adapted severe acute respiratory syndrome coronavirus 2 (SARS-CoV-2) (MA10) evoked kidney injury. BALB/c mice were nasally infected with MA10. *A* and *B*: serum creatinine levels and the urinary albumin/creatinine ratio (ACR) were measured on *day 4* after MA10 infection. Data are shown as means ± SD (*n* = 8–10); *P < 0.05 and **P < 0.01 by Student’s *t* test (*A*) or Mann–Whitney *U* test (*B*). *C*: kidney sections were stained with hematoxylin and eosin (H&E), and the severity of tubular injury was evaluated. Scale bars: 100 μm (low magnification images) and 30 μm (high magnification images). Data are shown as means ± SD (*n* = 5); *P < 0.05 by Welch’s *t* test. *D*: urinary neutrophil gelatinase‐associated lipocalin levels (NGAL)/creatinine ratio was measured on *day 4* after the MA10 infection. Data are shown as means ± SD (*n* = 8–10); *P < 0.05 by Mann–Whitney *U* test. *E*–*G*: expression of *Ifn-γ*, *Il-6*, and *Tnf-α* was examined on *day 4* after MA10 infection using quantitative PCR. Transcript expression was normalized to that of *Gapdh*. Data are shown as means ± SD (*n* = 5).

To examine the pathogenesis of COVID-19 in a murine model, BALB/c mice were nasally infected with MA10. MA10 infection led to 20% body weight loss on *day 4* (Fig. 1*A*). Hematoxylin and eosin staining indicated that the alveoli were disrupted, and infiltrating cells were increased in the lung of MA10-infected mice (Fig. 1*B*). Consistently, quantitative PCR analysis revealed that the mRNA expression of inflammatory cytokines, including IFN-γ, IL-6, and TNF-α, was upregulated in the lungs of MA10-infected mice (Fig. 1, *C*–*E*), suggesting that MA10 infection caused lung injury. Subsequently, we investigated whether kidney injury was induced by MA10 infection. Serum creatinine (sCr) levels and ACR were significantly higher in the MA10 infection group than in the noninfection group (Fig. 2, *A* and *B*). Histological analysis revealed that morphological abnormalities were not observed in the glomeruli of most mice. However, some proximal tubules exhibited degenerative changes characterized by swollen and vacuolated cytoplasms after MA10 infection (Fig. 2*C*). NGAL/Cr levels, a tubular injury marker, were significantly upregulated in the urine of MA10-infected mice (Fig. 2*D*). Unlike the pulmonary pathology, no upregulation of IFN-γ, IL-6, or TNF-α mRNA was observed in the kidneys of MA10-infected mice (Fig. 2, *E*–*G*). Collectively, MA10 infection resulted in kidney injury in mice.

### Cytokine Signaling May Affect Renal Homeostasis in MA10-Infected Mice

**Figure 3.**
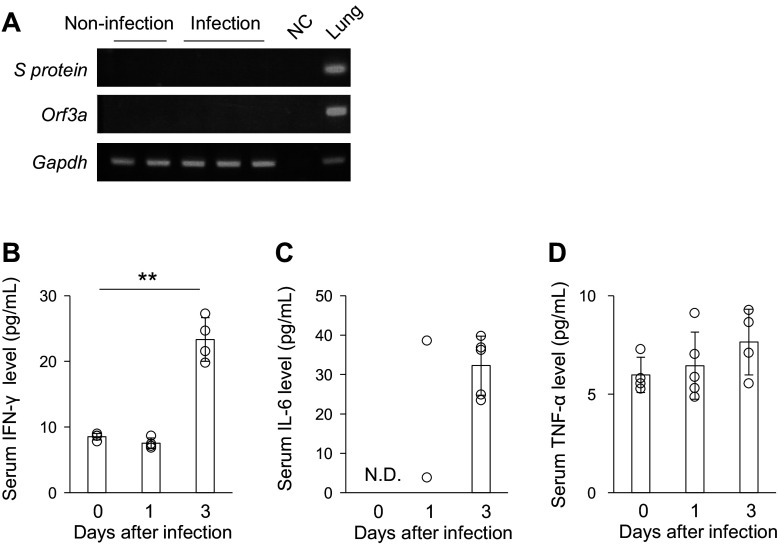
Inflammatory cytokines were upregulated in mice sera after mouse-adapted severe acute respiratory syndrome coronavirus 2 (SARS-CoV-2) (MA10) infection**.** *A*: PCR using specific primers for the detection of *Spike protein* and *Orf3a* was performed to examine MA10 expression in the kidneys on *day 4* after MA10 infection. Lung samples from MA10-infected mice were used as positive controls. *Gapdh* expression was used as an internal control. *B*–*D*: protein levels of interferon (IFN)-γ, interleukin (IL)-6, and tumor necrosis factor (TNF)-α in mouse sera were examined using ELISA after MA10 infection. Data are shown as means ± SD (*n* = 4–5); **P < 0.01 by Dunnett’s test.

Two issues remain unclarified regarding the mechanism underlying kidney injury in COVID-19: whether SARS-CoV-2 directly infects kidney cells and whether the SARS-CoV-2-induced cytokine storm indirectly affects kidney cells. First, PCR analysis was performed with two kinds of specific primer for the detection of MA10 (Fig. 3*A*). MA10 was not preferentially detected in the kidneys on *day 4* after infection, unlike in the lungs. In subsequent experiments, MA10 was detected only in the kidneys of some mice (MA10 + vehicle group in Fig. 5, *D* and *E*). Next, we evaluated the cytokine levels in the sera of MA10-infected mice using ELISA (Fig. 3, *B*–*D*). The protein levels of IFN-γ and IL-6 were significantly increased in the sera on *day 3* after MA10 infection, whereas those of TNF-α were not. These data suggest that the SARS-CoV-2-induced cytokine storm affects renal homeostasis, including the pathogenesis of kidney injury.

### Inhibition of Antiviral Activity During Early MA10 Infection Exacerbated Kidney Injury

**Figure 4.**
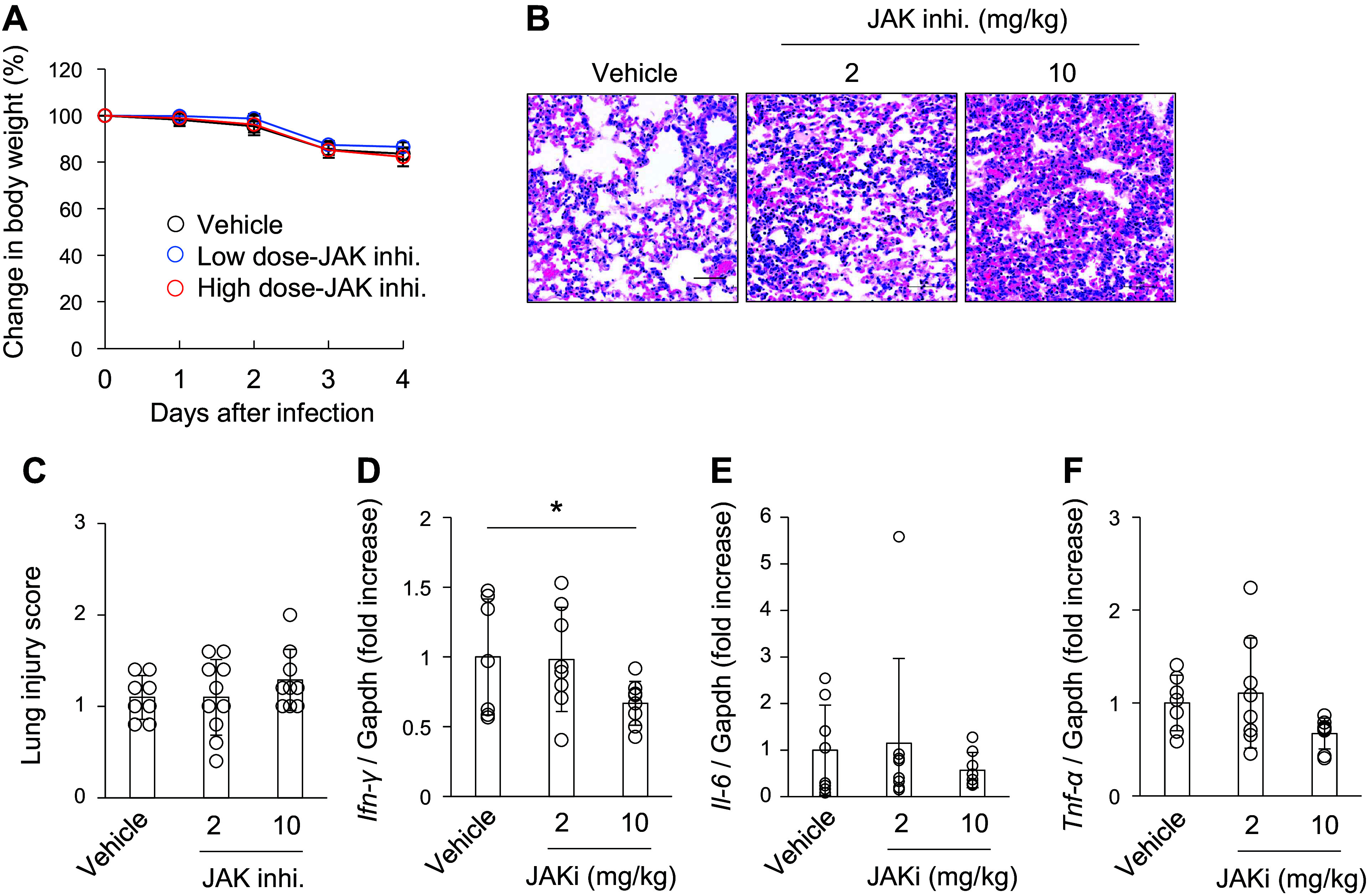
Janus kinase (JAK) inhibition on *days 1*–*3* after mouse-adapted severe acute respiratory syndrome coronavirus 2 (SARS-CoV-2) (MA10) infection did not significantly affect lung injury. MA10-infected mice were administered the JAK inhibitor baricitinib on *days 1*–*3*. *A*: body weight changes were measured after MA10 infection. *B* and *C*: hematoxylin and eosin (H&E) staining of murine lungs after MA10 infection. Scale bars: 50 μm. The severity of lung injury scores is also shown. *D*–*F*: expression of *Ifn-γ*, *Il-6*, and *Tnf-α* was examined on *day 4* after MA10 infection using quantitative PCR. Transcript expression was normalized to that of *Gapdh*. Data are shown as means ± SD (*n* = 8–9); *P < 0.05 by Dunnett’s test.

IFNs/JAK signaling exhibits antiviral activity against RNA viruses. Mice were treated with a JAK inhibitor baricitinib (2 mg/kg and 10 mg/kg) on *days 1*–*3* after MA10 infection to investigate the pathological mechanisms of MA10-induced kidney injury. The low dose of 2 mg/kg corresponds to that used in humans based on the AUC of baricitinib in rats (20–22). The high dose (10 mg/kg) was used to evaluate the pharmacological effects of baricitinib, as described in previous reports (23, 24). Changes in body weight were similar between the JAK inhibitor and vehicle groups (Fig. 4*A*). Histological analysis revealed that baricitinib increased lung inflammation; however, the difference was not statistically significant (Fig. 4, *B* and *C*). High-dose baricitinib reduced IFN-γ expression in the lungs, whereas other inflammatory cytokines were unchanged (Fig. 4, *D*–*F*).

**Figure 5.**
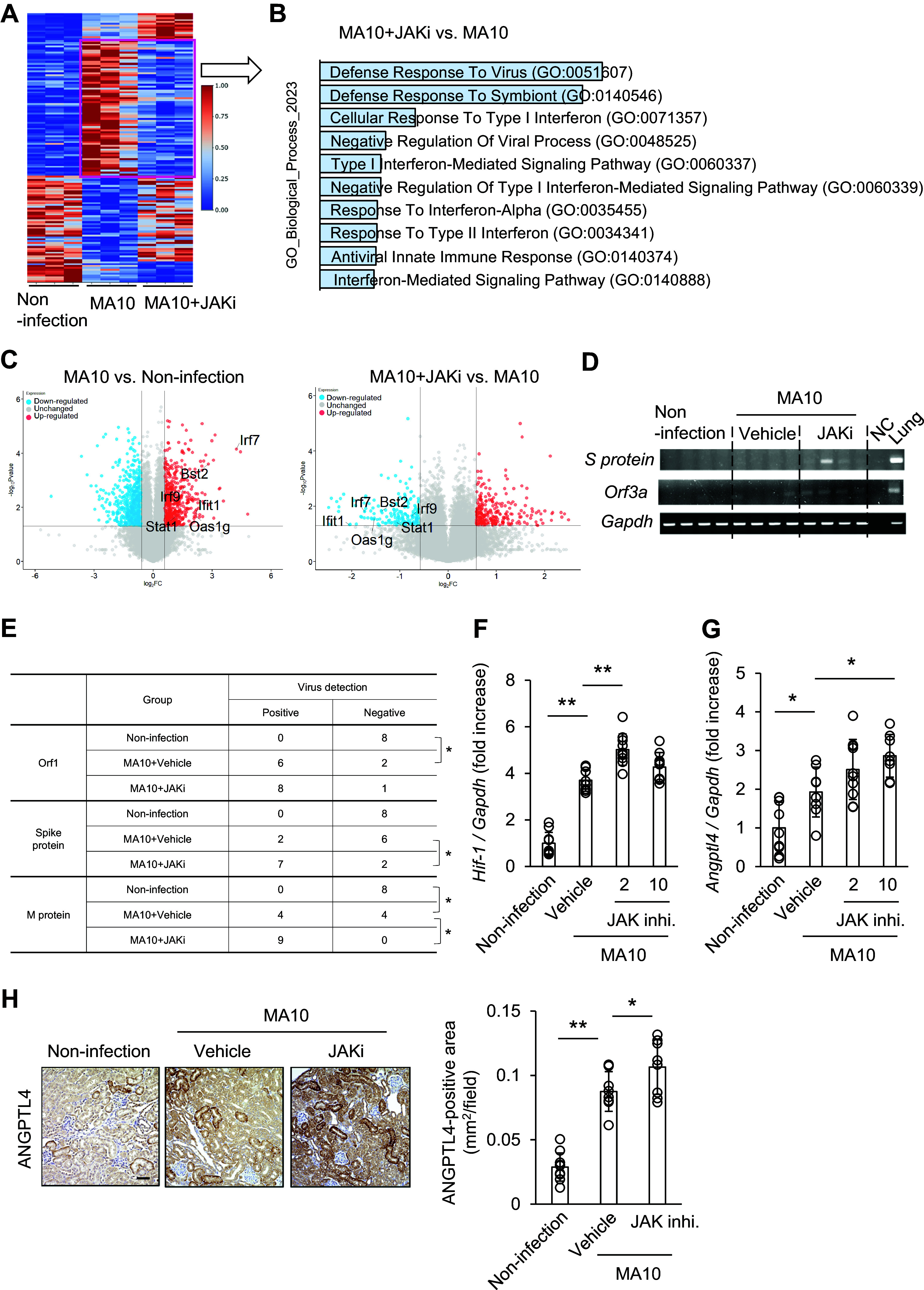
Janus kinase (JAK) inhibition on *days 1*–*3* after mouse-adapted severe acute respiratory syndrome coronavirus 2 (SARS-CoV-2) (MA10) infection suppressed the intrinsic antiviral activity of interferons**.** MA10-infected mice were administered the JAK inhibitor baricitinib on *days 1*–*3*. RNA-sequencing was performed using the kidneys of noninfected and MA10-infected mice treated with the vehicle [0.5% carboxymethyl cellulose (CMC)] or 10 mg/kg baricitinib. *A*: heatmap showing the proportion of genes with >1.5-fold change between noninfected and MA10-infected mice and between MA10 and MA10 + JAKi with *P* < 0.05. *B*: Gene Ontology analysis of genes whose expression decreased 1.5-fold following JAK inhibitor treatment. *C*: volcano plots showing representative genes associated with interferon (IFN) signaling. Bst2, bone marrow stromal cell antigen 2; Irf, interferon regulatory factor; Ifit1, interferon induced protein with tetratricopeptide repeats 1; Oas1, 2′-5′-oligoadenylate synthetase 1; Stat1, signal transducer and activator of transcription 1. *D*: PCR of *S protein* and *Orf3a* in the kidneys. Lung samples from MA10-infected mice were used as positive controls. *Gapdh* expression was used as an internal control. Representative images are shown. *E*: quantitative PCR was performed to detect *Orf1*, *S protein*, and *M protein* in the kidneys. The number of mice in which MA10 was detected in the kidneys is shown. *P < 0.05 by χ^2^ test. *F* and *G*: expression of *Hif-1 and Angptl4* was examined on *day 4* after MA10 infection using quantitative PCR. Transcript expression was normalized to that of *Gapdh*. Data are shown as means ± SD (*n* = 8–10); *P < 0.05 and **P < 0.01 by Dunnett’s test. *H*: immunohistochemistry was performed to detect angiopoietin-like (ANGPTL)4 protein levels. Representative images are shown. Scale bar: 50 μm. The expression level was measured. Data are shown as means ± SD (*n* = 8–10); *P < 0.05 and **P < 0.01 by Dunnett’s test.

RNA-sequencing was performed using the kidneys of noninfected mice and MA10-infected mice treated with the vehicle (MA10) or high-dose baricitinib (MA10 + JAKi). The proportion of genes with a fold change between noninfected and MA10-infected mice and between MA10 and MA10 + JAKi greater than 1.5 and *P* < 0.05 is shown in Fig. 5*A*. The renal pathophysiological feature was not identified by the GO analysis of genes whose expression was downregulated by MA10 infection and upregulated by the JAK inhibitor (Supplemental Fig. S1). GO analysis of genes whose expression decreased 1.5-fold following JAK inhibitor treatment and volcano plots showed that IFN signaling-associated genes were preferentially altered by MA10 infection and JAKi treatment (Fig. 5, *B* and *C*). IFN signaling-associated genes, such as bone marrow stromal cell antigen 2 (Bst2), interferon regulatory factor 7 (Irf7), Irf9, IFN-induced protein with tetratricopeptide repeats 1, 2′-5′-oligoadenylate synthetase 1g (Oas1g), and signal transducer and activator of transcription 1 were upregulated by MA10 infection and downregulated by treatment with JAKi. Low IFN expression correlates with COVID-19 severity (25), and IFN signaling exhibits intrinsic antiviral activities against RNA viruses (14). Therefore, we hypothesized that intrinsic antiviral activities were suppressed in the kidneys of JAKi-treated mice. PCR and qPCR analyses were performed to detect the presence of MA10 using various MA10-specific primers. The MA10 + JAKi group exhibited higher incidence of MA10 in the kidneys than the MA10 group (Fig. 5, *D* and *E*). Angiopoietin-like 4 (ANGPTL4) is induced by hypoxia as well as hypoxia-inducible factor 1 (HIF-1) (26), and is a novel marker of COVID-19 severity (27). qPCR revealed that the mRNA expression of *Hif-1* and *Angptl4* was upregulated in the kidneys of MA10-infected mice and further upregulated by the JAK inhibitor (Fig. 5, *F* and *G*). The ANGPTL4 protein level was also upregulated in the kidneys of JAKi-treated mice compared with the vehicle group (Fig. 5*H*). These results indicate that JAK inhibition also promoted a hypoxia response in the kidneys of MA10-infected mice. However, it is unclear whether the hypoxia response can be attributed to a decrease in endogenous antiviral activity or a decrease in blood oxygen concentration.

**Figure 6.**
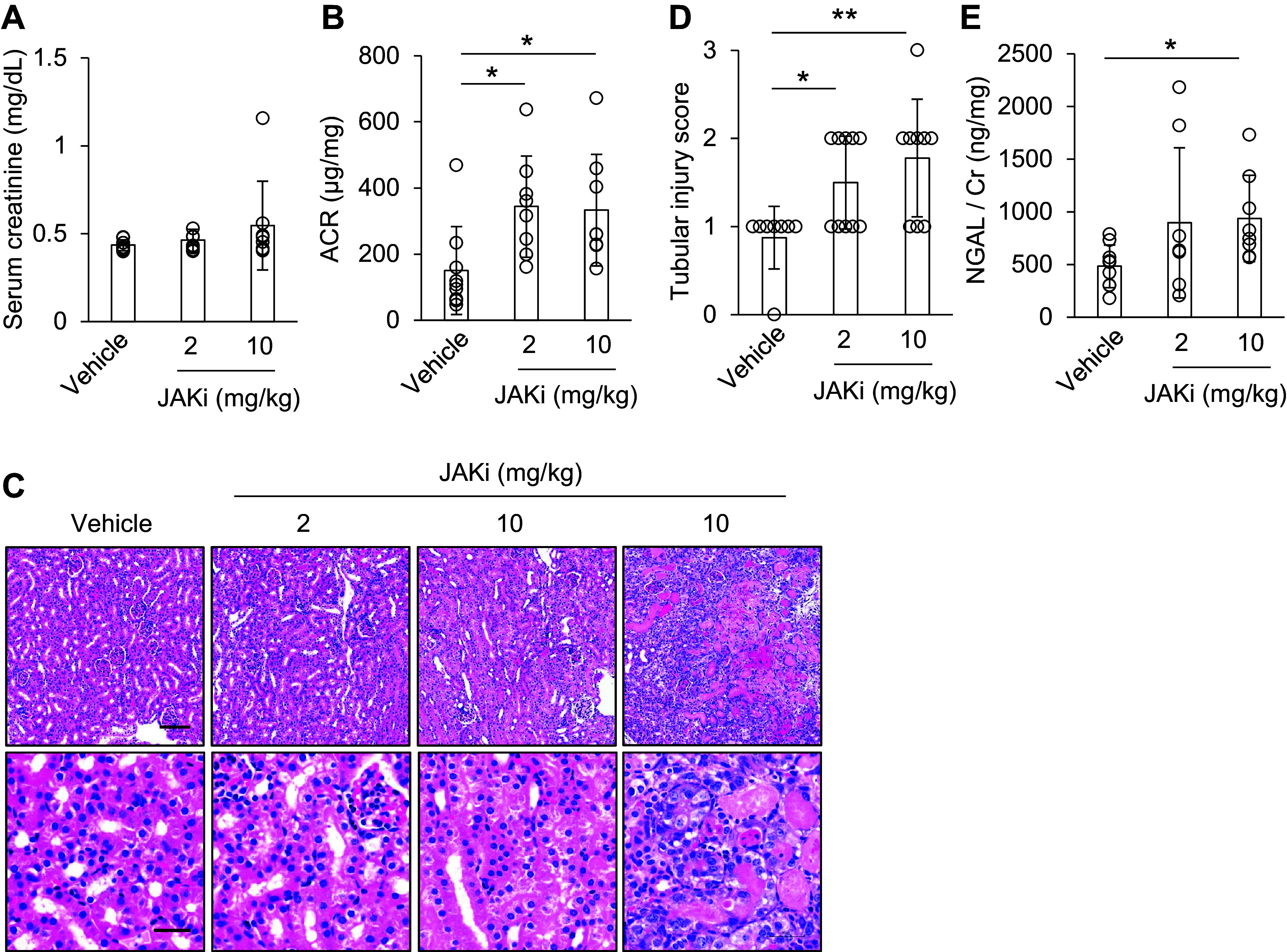
Janus kinase (JAK) inhibition on *days 1*–*3* after mouse-adapted severe acute respiratory syndrome coronavirus 2 (SARS-CoV-2) (MA10) infection exacerbated kidney injury**.** MA10-infected mice were administered the JAK inhibitor baricitinib on *days 1*–*3*. *A* and *B*: serum creatinine levels and albumin-to-creatinine ratio (ACR) were measured on *day 4* after MA10 infection. *C* and *D*: hematoxylin and eosin (H&E) staining of murine kidneys after MA10 infection. Scale bars: 100 μm (low magnification) and 30 μm (high magnification). The severity of tubular damage scores is also shown. *E*: neutrophil gelatinase‐associated lipocalin levels (NGAL)/Cr ratio was measured on *day 4* after MA10 infection using ELISA. Data are shown as means ± SD (*n* = 8–10); *P < 0.05 and **P < 0.01 by Dunnett’s test.

Notably, sCr levels were unaffected by baricitinib, whereas the ACR was higher in the baricitinib group than in the vehicle group (Fig. 6, *A* and *B*). Baricitinib increased tubular degeneration and the NGAL/Cr ratio, especially at high doses (Fig. 6, *C*–*E*). The treatment with high-dose baricitinib induced tubular necrosis in one of eight mice. This indicated that baricitinib exacerbated tubular injury in MA10-infected mice. In addition, high-dose baricitinib did not induce kidney injury markers in noninfected mice (Supplemental Fig. S2). Intriguingly, according to the effect of MA10 infection and JAK inhibition on the liver, JAK inhibition did not affect the levels of MA10 infection-induced liver injury marker aspartate aminotransferase (Supplemental Fig. S3). These findings indicate that intrinsic antiviral activity contributes to protection against MA10 infection-induced kidney injury.

## DISCUSSION

In this study, we demonstrate that intranasal infection with mouse-adapted SARS-CoV-2 MA10 in mice induced kidney injury. Treatment with the JAK inhibitor baricitinib exacerbated kidney injury, suggesting that cytokine release in response to MA10 infection may affect renal homeostasis. Moreover, this exacerbation was accompanied by suppression of the intrinsic antiviral activity of IFNs. Our findings provide the first evidence of the impact of early JAK inhibition during SARS-CoV-2 infection on kidneys in vivo.

To comprehend the pathogenesis of COVID-19 and develop novel therapeutic strategies, animal models of COVID-19 are crucial. Conventional laboratory mice strains cannot be used to examine the pathogenesis of human SARS-CoV-2 infection because of the lack of binding between the S protein of human SARS-CoV-2 and mouse ACE2. Previous studies have utilized mice infected with MA10 or human ACE2 transgenic/knock-in mice (28–30). Our preliminary experiments indicated that SARS-CoV-2 infection only minimally affected the body weight of human ACE2 knock-in mice (data not shown). Consequently, we utilized MA10-infected mice as a model to mimic COVID-19 infection.

Clinically, patients often present with AKI or abnormal urinary findings after SARS-CoV-2 infection (2, 3). Tubular injury is a common feature of AKI observed in patients with COVID-19 (31). MA10-infected mice exhibited increased tubular cells with vacuoles and tubular injury marker NGAL/sCr. In addition, ACR was elevated in MA10-infected mice, indicating similarities between the characteristics of kidney damage in MA10-infected mice and the clinical picture. At the beginning of the COVID-19 pandemic, researchers speculated that SARS-CoV-2 could directly infect the kidneys and cause damage (32, 33), given the expression of ACE2 in tubular cells and podocytes (6, 7). However, SARS-CoV-2 was not detected in kidney biopsy samples (10), and some research groups found no virus in the kidneys of COVID-19 animal models (28, 29). In this study, MA10 was not preferentially detected in the kidneys by PCR. Moreover, the expression of cytokines and hypoxia-related molecules was upregulated in MA10-infected mice, leading us to speculate that indirect effects induced by MA10 infection, such as cardiocirculatory failure and cytokine storm, cause kidney injury.

IFNs exhibit antiviral activities against RNA viruses, including SARS-CoV-2 through JAK1/2. For example, BST2 (also termed tetherin or CD317), an IFN-inducible type II transmembrane glycoprotein, restricts SARS-CoV-2 replication (34). *Bst2* mRNA expression was upregulated by MA10 infection and downregulated in the kidneys of the MA10 + JAKi group compared with those of the MA10 group. These findings are consistent with previous reports showing a correlation between low IFN expression and the severity of COVID-19 (25), and that IFN activation confers resistance to SARS-CoV-2-related kidney damage (35). However, baricitinib, a JAK inhibitor acting on JAK1/2 identified as a candidate medicine using artificial intelligence (36), is used for the treatment of severe pneumonia in patients with COVID-19, together with remdesivir, an antiviral medicine (13, 37). A recent study revealed that increased endogenous retroviruses contribute to renal fibroinflammation through the upregulation of IFN-related genes, such as retinoic acid-inducible gene I, melanoma differentiation-associated gene 5, IFN response genes, and IFN-stimulated genes (38). These genes are also activated in SARS-CoV-2-infected cells (39). In addition, JAK inhibition prevents COVID-19 cytokine-induced injury in human organoid-derived podocytes (16), suggesting its therapeutic potential against kidney injury in patients with COVID-19. The median time from symptom onset to the random assignment of baricitinib with remdesivir was eight days in the ACTT-2 study (40), indicating that these two drugs were administered in the late phase of infection. Therefore, JAK inhibition combined with antiviral therapy during the late stage of COVID-19 may be useful for the prevention of kidney damage.

Baricitinib is commonly used for the treatment of rheumatoid arthritis. In this study, although baricitinib administration did not increase sCr, ACR and NGAL were significantly increased in JAKi-treated mice infected with MA10 as a clinical manifestation of AKI. Taken together with previous reports that the disease activity of rheumatoid arthritis is associated with COVID-19 severity (41, 42), this suggests that patients with rheumatoid arthritis and COVID-19 should be treated carefully, especially when the patients are administered JAK inhibitors.

In conclusion, antiviral activity in the kidneys through JAK kinases exhibits resistance to injury during the early stage of MA10 infection. These findings provide novel insights into the pathogenesis of COVID-19-associated nephropathy and may contribute to more informed prescription practices for the treatment of COVID-19.

## DATA AVAILABILITY

Data supporting the findings of the present study are available from the corresponding author, M.O., upon reasonable request. RNA-sequencing data have been deposited in the Gene Expression Omnibus and are available under Accession No. GSE249303.

## SUPPLEMENTAL DATA

10.6084/m9.figshare.25521754 Supplemental Figs. S1–S3 and Supplemental Table S1: https://doi.org/10.6084/m9.figshare.25521754.

## GRANTS

This work was partially supported by the Japanese Association of Dialysis Physicians (to M.O.), the Hoansha Foundation (to M.O.), the Mochida Memorial Foundation for Medical and Pharmaceutical Research (to M.O.), Japan Agency for Medical Research and Development (AMED) Grants JP23ama121052 and JP23ama121054, and the Nippon Foundation-Osaka University Project for Infectious Disease Prevention.

## DISCLOSURES

N.T. and Y.Y. are employees of the Research Foundation for Microbial Diseases of Osaka University. None of the other authors has any conflicts of interest, financial or otherwise, to disclose.

## AUTHOR CONTRIBUTIONS

Y.Y., Y.F., and M.O. conceived and designed research; H.S., H.K., N.T., S.Tamiya, C.O., Y.M., and M.O. performed experiments; H.S., H.K., T.I., A.Y., S.Tanaka, D.O., Y.O., and Y.F. analyzed data; H.S. and M.O. interpreted results of experiments; H.S., H.K., and M.O. prepared figures; H.S., Y.O., Y.Y., Y.F., and M.O. drafted manuscript; H.S., Y.O., Y.Y., Y.F., and M.O. edited and revised manuscript; H.S., H.K., N.T., T.I., S.Tamiya, A.Y., S.Tanaka, D.O., C.O., Y.M., Y.O., Y.Y., Y.F., and M.O. approved final version of manuscript.
